# Ring Avulsion Digital Replantation Utilizing a Cannulated Axial Screw for Bone Fixation

**DOI:** 10.1097/GOX.0000000000002352

**Published:** 2019-08-05

**Authors:** Alessandro Thione, Alberto Sánchez-García, Alberto Pérez-García, Enrique Salmerón-González, Elena García-Vilariño

**Affiliations:** From the Department of Plastic and Reconstructive Surgery, University Hospital La Fe, Valencia, Spain

## INTRODUCTION

Ring avulsion injuries are quite uncommon, despite the small forces needed to produce a complete amputation.^1^ According to our experience, replantation is particularly challenging in this type of injuries due to the extent of damage sustained by skin, nerves, and vessels, but recommended because of good expected functional results.

Several bone fixation methods have been described in digital replantation, such as single or double K-wire fixation, or intraosseus wire, obtaining variable results in terms of surgical time requirements, fixation stability, infection rates, and reintervention for osteosynthesis material removal.^2^ Our aim is to present the case of a patient on whom bone fixation was made by utilizing a partially threaded cannulated axial screw, which allows a one-stage surgical procedure in digital replantation. This type of fixation, which has been utilized for interphalangeal arthrodesis and finger fractures treatment, confers a realiable bony stability.^3^ This allows for early motion of the digit without having to wait for complete bony healing. Those factors may help to alleviate stiffness and allow prompt recovery of finger functionality.

## CASE REPORT

**Fig. 1.**
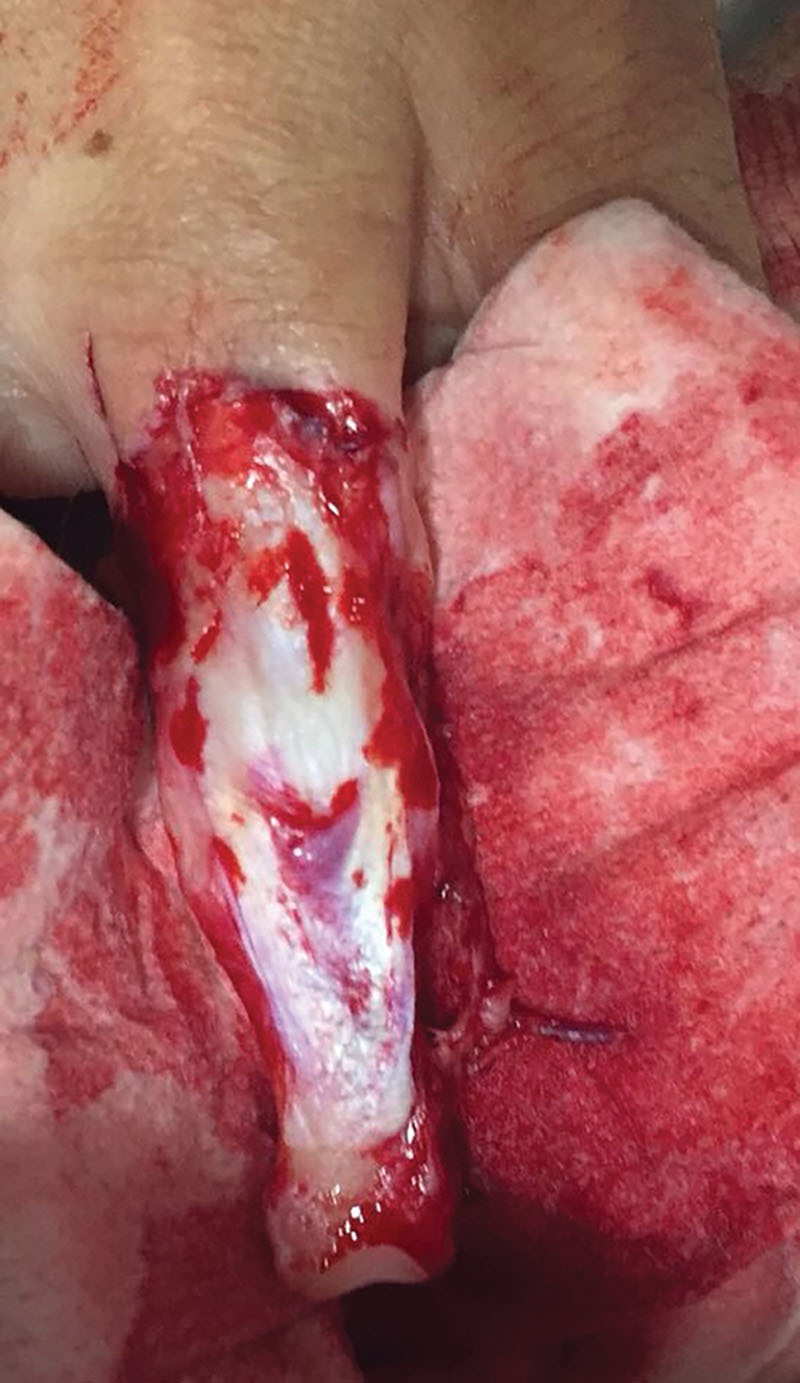
Total circumferential soft tissue degloving, associated with distal phalanx bone loss at distal interphalangeal joint level.

A 32-year-old male patient was referred to our center presenting a ring avulsion injury on the fourth finger of left nondominant hand, in the context of a sport accident. The lesion consisted of a total circumferential soft tissue degloving starting from proximal phalanx, associated with distal phalanx bone loss at interphalangeal joint level (Fig. 1). Ring amputation was classified as type III, according to Urbaniak et al.^4^ The patient arrived during the morning, with 4 hours of cold ischemia time, so was transferred to the operating theater.^5^

Replantation was successfully performed according to a protocolized sequence: soft tissue debridement, neurovascular pedicles identification in amputated part and stump, bone fixation, 2 dorsal vein anastomosis, radial collateral artery anastomosis using a venous by pass—tunnelized through the amputated skin—and neurorraphy.

**Fig. 2.**
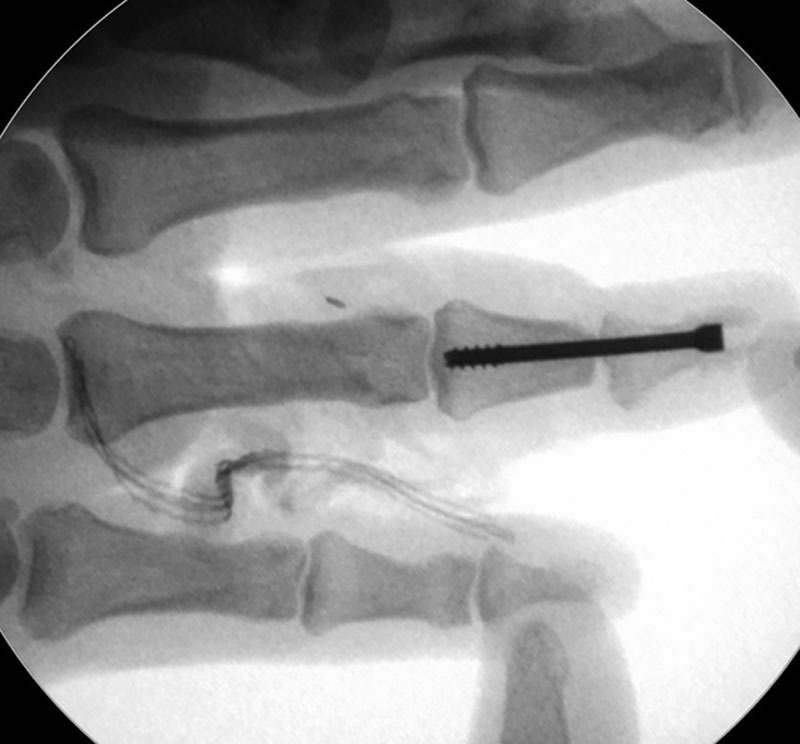
Intraoperative radiograph showing 2-mm axial screw fixation, achieving satisfactory anatomic distal phalanx alignment.

In this case, bone fixation was performed using a 2-mm partially threaded cannulated axial screw (AO-Synthes, West Chester, Pennsylvania) through distal and middle phalanx, obtaining required stabilization (Fig. 2).

**Fig. 3.**
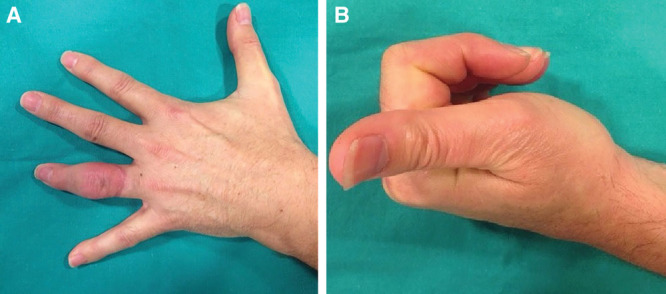
Four-week postoperative image showing A, reimplanted finger viability and B, complete hand gripping.

The postoperative period was uneventful and rehabilitation started at postoperative day 10, moving all the fingers and above all proximal interphalangeal joints. The patient presented a rapid recovery process, allowing preservation of complete flexo-extension of proximal interphalangeal joint, and a proper function of the fourth finger after the first month (Fig. 3).

## DISCUSSION

Stable bone fixation must be achieved before the repair of other structures in the digital reimplantation process.^6^ Numerous methods for osteosynthesis have been described in the literature, such as crossed Kirschner wires, a combination of intraosseus cerclage wires and Kirschner wires, and intramedullary Kirschner wires.^2,7^ However, most of those require a subsequent surgical intervention for osteosynthesis material removal, which may delay rehabilitation process. In our patient, axial screw bone fixation allowed early active range-of-motion exercises, which had a substantial effect on flexibility, dexterity, and strength of patient’s replanted finger and hand function.

According to us, bone fixation in digital reimplantation by this method has a determinant advantage: it allows carrying out a reliable and stable fixation, which would allow for early motion of the digit. This is due to both the intrinsic stability provided by this method of fixation and the unneeded osteosynthesis material removal. Thus, satisfactory functional results can be rapidly achieved.

## SUMMARY

Finger replantation in ring avulsion injuries may be particularly challenging. A stable bone fixation is required previously to neurovascular reparation. Several bone fixation methods have been described in digital replantation, such as single or double K-wire fixation, or intraosseus wire. This case report illustrates the utilization of a partially threaded cannulated axial screw, which provides reliable bony stability, and allows a one-stage surgical procedure in digital replantation. Those factors allow for early motion of the digit and allow prompt recovery of finger functionality.

## References

[1] Kupfer DM Eaton C Swanson S Ring avulsion injuries: a biomechanical study. J Hand Surg Am. 1999;24:1249–1253. 10584949 10.1053/jhsu.1999.1249

[2] Lee SW Lee DC Kim JS Analysis of bone fixation methods in digital replantation. Arch Plast Surg. 2017;44:53–58. 28194348 10.5999/aps.2017.44.1.53 PMC5300925

[3] Beldner S Polatsch DB Arthrodesis of the metacarpophalangeal and interphalangeal joints of the hand: current concepts. J Am Acad Orthop Surg. 2016;24:290–297. 27097126 10.5435/JAAOS-D-15-00033

[4] Urbaniak JR Evans JP Bright DS Microvascular management of ring avulsion injuries. J Hand Surg Am. 1981;6:25–30. 7204914 10.1016/s0363-5023(81)80006-8

[5] Cavadas PC Rubí C Thione A Immediate versus overnight-delayed digital replantation: comparative retrospective cohort study of survival outcomes. J Hand Surg Am. 2018;43:625–630. 29751978 10.1016/j.jhsa.2018.03.047

[6] Tupper JW Techniques of bone fixation and clinical experience in replanted extremities. Clin Orthop Relat Res. 1978;165–168. 688705

[7] Touliatos AS Soucacos PN Beris AE Alternative techniques for restoration of bony segments in digital replantation. Acta Orthop Scand Suppl. 1995;264:19–22. 7604723 10.3109/17453679509157159

